# Prognostic value of serum α-HBDH levels in patients with lung cancer

**DOI:** 10.1186/s12957-023-02965-3

**Published:** 2023-03-06

**Authors:** Zhi-Min Yuan, Long-Hao Wang, Cheng Chen

**Affiliations:** 1 Department of Clinical Laboratory, Shaanxi Provincial Cancer Hospital, Xi’an, Shaanxi China; 2 Department of Otorhinolaryngology‑Head and Neck Surgery, Shanghai Ninth People’s Hospital, Shanghai Jiaotong University, Shanghai, China; 3 Department of General Dentistry/Key Laboratory of Shaanxi Province for Craniofacial Precision Medicine Research, College of Stomatology, Xi’an Jiaotong University, Xiwu Road 98# Xi’an, Shaanxi, 710004 China

**Keywords:** α-HBDH, Lung cancer, Survival time, Prognosis

## Abstract

**Background:**

The purpose of our study is to investigate the expression level and prognostic value of serum α-hydroxybutyrate dehydrogenase (α-HBDH) in lung cancer (LC) patients.

**Method:**

LC patients treated in the Department of Oncology, Shaanxi Provincial Cancer Hospital from January 2014 to December 2016 were included in this study, all of whom underwent serological detection of α-HBDH prior to admission, and were enrolled in follow-up 5-year survival. Comparing the differences between high group and normal groups based on α-HBDH and LDH expression via clinicopathological parameters and laboratory data. Univariate and multivariate regression and overall survival (OS) were analyzed to explore whether elevated α-HBDH was an independent risk factor for LC, compared to LDH.

**Results:**

Multivariate regression analysis showed that age (*P* = 0.018), liver metastasis (*P* = 0.011), α-HBDH (*P* = 0.015), and neutrophil-to-lymphocyte ratio (NLR) (*P* = 0.031) were independent prognostic factors affecting OS in LC patients. The overall diagnostic efficacy of α-HBDH (AUC = 0.887) was higher than that of LDH (AUC = 0.709) in the ROC curve. The sensitivity was significantly higher of α-HBDH (sensitivity: 76.06%, specificity: 94.87%) compared with LDH (sensitivity: 49.30%, specificity: 94.87%). The median of OS was more significant in the high-α-HBDH group (6.4 months) than in the normal-α-HBDH group (12.7 months) (*P* = 0.023). The median of OS was significant in the high-LDH (> 245 U/L) group at 5.8 months and 12.0 months in the normal-LDH (≤ 245 U/L) group (*P* = 0.068).

**Conclusions:**

Elevated expression of α-HBDH may indicate a poor prognosis of LC patients. It has a higher sensitivity than LDH and can be used as a potential early biomarker and an independent risk factor predicting the prognosis of LC survival.

**Supplementary Information:**

The online version contains supplementary material available at 10.1186/s12957-023-02965-3.

## Introduction

Serum α-hydroxybutyrate dehydrogenase (α-HBDH) is an isoenzyme of lactate dehydrogenase (LDH), and its acts by catalyzing the oxidation of α-hydroxybutyrate to α-ketobutyric acid [1]. In the human body, α-HBDH is found in a variety of tissues, with the highest concentration in the cardiac tissue, followed by the red blood cells, white blood cells, and kidneys [2–4]. Studies have reported and confirmed that α-HBDH is significantly upregulated in many diseases and is associated with severe adverse prognoses, such as core-binding factor-related acute myeloid leukemia [5], acute pancreatitis [6], liver injury [7], and AIDS [8], which can be used as a potential early biomarker and an early prognostic indicator.

When cardiomyocytes are damaged, hydroxybutyrate dehydrogenase is released into the serum in large quantities, so it can be used as an indicator of myocardial damage [9]. Subsequently, researchers found that some patients with acute and chronic malignant tumors had abnormally elevated serum α-HBDH levels. It has been observed that serum α-HBDH is significantly elevated in patients with some hematological tumors [10] and malignant ovarian tumors [11]. Early scholars found that intracranial tumors can also lead to the abnormal elevation of α-HBDH and concluded that it may be used as an indicator of poor prognosis [12]. Khanolkar et al. study found that serum α-HBDH levels were elevated in patients with testicular tumors than in healthy subjects [13]. Therefore, we believe that this index may also be increased in malignant tumors, but there are few studies in this area.

Lung cancer (LC) is still one of the major malignant tumors that seriously endanger human health. Few studies have assessed the value of serological α-HBDH as a predictor and prognostic indicator in LC patients [14]. Therefore, we hypothesized that serum α-HBDH is associated with LC prognosis and explored the value of α-HBDH for prognosis in LC.

However, there are some studies on the abnormal expression of LDH in tumors, and many scholars take it as one of the prognostic indicators [15, 16]. Existing studies have demonstrated that elevated LDH in tumor tissue is associated with the clinical outcomes of multiple cancers, including renal cell carcinoma, melanoma, prostate cancer, lung cancer, and colorectal cancer [17, 18]. Could α-HBDH also be a biomarker for the prognosis and diagnosis of lung cancer? How are its sensitivity and specificity compared to LDH? There are also few comparing studies on the specificity and sensitivity of α-HBDH and LDH expression as biomarkers in the serum of tumor patients [13, 19]. So this study also compared the specificity and sensitivity of the α-HBDH and LDH, providing data support for the biological indicators of tumor prediction and prognosis of LC patients.

## Materials and methods

### Study population

This study was approved by the Ethics Committee of Shaanxi Provincial Cancer Hospital (No. 2021072), and the ethics committee approved an exemption from informed consent for this study. All methods were carried out in accordance with relevant guidelines and regulations. Collected LC patients with serum α-HBDH and LDH test results were hospitalized in Shaanxi Provincial Cancer Hospital from January 2014 to December 2016, excluded other clinical diseases associated with abnormal α-HBDH and LDH, such as cardiac disease, muscular dystrophy, vitamin B12 deficiency, hemolytic anemia, renal infarction, renal vascular embolization, and cachexia. Finally, we obtained 71 eligible LC patients, including 52 males and 19 females, aged between 39 and 87 years. Clinic pathological data were obtained from patients’ electronic medical records, which included age, gender, smoking history, tumor stage, lymph nodes, and metastasis of other sites (liver, kidney, brain, and bone), clinical routine, and biochemical and tumor markers.

### Follow-up

This study logged into the medical record system of Shaanxi Cancer Hospital and obtained the basic information and general information of patients by consulting the inpatient history. In addition, medical records were screened according to the aforementioned inclusion and exclusion criteria. The screened cases were followed up. Through telephone follow-up and consulting the follow-up table, we obtained the main contents of the follow-up, involving the time of death, cause of death, and current living conditions. The study follow-up deadline was December 31, 2021. Cases affecting the results of the study, such as deaths due to accidents or other diseases, were excluded based on the exclusion criteria and the cause of death obtained. All the families included in the study were informed that their cases had been included in a retrospective study and had passed ethical review.

### Data collection

General data including gender, age, tumor type, stage, and metastasis were collected. Hematological data were collected at pre-admission examination, including α-HBDH, LDH, homocysteine (HCY), fibrinogen (FIB), carcinoembryonic antigen (CEA), alpha-fetoprotein (AFP), total protein (TP), albumin (ALB), total calcium (Ca), leukocyte (WBC), neutrophil-to-lymphocyte ratio (NLR), and platelets (PLT) values. All patients’ electronic medical records and laboratory test results were reviewed by an independent physician.

The α-HBDH, LDH, HCY, TP, ALB, and Ca were analyzed by AU5800 Fully Automatic Biochemical analyzer from Beckman Coulter (American) Co., Ltd., WBC and PLT were analyzed by BC-6800 Fully Automatic Serum Cell analyzer from Mindray Medical International (Shenzhen) Co., Ltd., AFP and CEA were analyzed by E411 Automatic Immunoanalyzer from Roche Diagnostic Products (Shanghai) Co., Ltd., and FIB was analyzed by STA-R Fully Automatic Hemagglutator from Stago Diagnostic Technology (Tianjin) Co., Ltd. During the test, quality control was carried out with the supporting quality control materials of the instruments.

### Statistics

In this study, categorical variables were described as proportions and were compared by using *χ*^2^ tests. Continuous variables were described as mean (*x* ± SD) or median (IQR). Comparisons of continuous variables between groups were done using *t* tests, one-way ANOVA, or equivalent non-parametric tests. The Cox proportional hazards regression model was used to perform the analysis α-HBDH with overall survival (OS). The ROC curves were evaluated for the diagnostic efficacy of the patient α-HBDH and LDH. The application SPSS 23.0 was used for all statistical comparisons, and the significant statistical level was set at the threshold of *p* < 0.05. The comparison of ROC curves and OS by GraphPad Prism 9.0 software (GraphPad Software, Inc.). The cutoff level of α-HBDH was set to a normal upper limit of 220 U/L, and the cutoff level of LDH was set to a normal upper limit of 245 U/L according to the reference range established in our laboratory.

## Results

### Clinical characteristics and baseline demographics of LC patients

Seventy-one patients with lung cancer were included in this study sequence. As shown in Table 1, the mean age of the study cohort was 64.2 years. Of the 71 patients, 52 were male and 19 were female. Twenty-five of the 71 patients (35%) had a history of smoking. Of the 71 patients, 45% (32) had lymph node metastases, 27% (19) had bone metastases, 18% (13) had brain metastases, 8 (11%) had liver metastases, and 3 (4%) had renal metastases. Among the 71 patients, 12 (17%) were in the low stage (stages I and II) and 59 (83%) were in the high stage (stages III and IV). Of the 71 patients, 17 (24%) were small cell lung cancer, 36 (51%) were lung adenocarcinoma, and 18 (25%) were squamous cell carcinoma. The average α-HBDH was 307 ± 152 U/L before admission.

**Table 1:** Comparison of clinical characteristics and laboratory data of serum α-HBDH in LC patients

Variables	Cases(*n* = 71)	α-HBDH > 220U/L (*n* = 44)	α-HBDH ≤ 220U/L (*n* = 27)	*p* value
Clinical characteristic				
Age (mean ± SD), years	64.2 ± 11.0	63.5 ± 11.8	65.3 ± 10.1	0.699
Gender				
Male	52 (73%)	28 (64%)	24 (89%)	0.021*
Female	19 (27%)	16 (36%)	3 (11%)	
Smoking	25 (35%)	15 (34%)	10 (37%)	0.802
Metastasis				
Liver	8 (11%)	6 (14%)	2 (7%)	0.640
Lymphnodes	32 (45%)	24 (55%)	8 (30%)	0.042*
Kindey	3 (4%)	2 (5%)	1 (4%)	0.865
Brain	13 (18%)	10 (23%)	3 (11%)	0.222
Bone	19 (27%)	15 (34%)	4 (15%)	0.077
Staging (I + II/III + IV)				
Low (I + II)	12 (17%)	5 (11%)	7 (26%)	0.096
High (III + IV)	59 (83%)	39 (89%)	20 (74%)	
Classification				
Small-cell carcinoma	17 (24%)	12 (27%)	5 (19%)	0.109
Adenocarcinoma	36 (51%)	24 (55%)	12 (44%)	
Squamous cell carcinoma	18 (25%)	8 (18%)	10 (37%)	
Laboratory data				
LDH, U/L	284 ± 135	351 ± 132	176 ± 32	< 0.001***
HCY, umol/L	27.6 ± 52.5	31.6 ± 66.3	21.2 ± 9.2	0.644
FIB, g/L	4.27 ± 1.51	4.34 ± 1.57	4.16 ± 1.42	0.603
CEA, ng/ml	22.3 ± 20.6	25.3 ± 23.3	17.3 ± 14.3	0.079
AFP, IU/ml	10.4 ± 6.3	11.2 ± 7.4	9.2 ± 3.5	0.141
TP, g/L	66.2 ± 8.01	65.5 ± 7.54	67.2 ± 8.75	0.404
ALB, g/L	36.1 ± 6.39	35.6 ± 5.79	36.9 ± 7.31	0.451
Ca, mmol/L	2.26 ± 0.25	2.24 ± 0.20	2.31 ± 0.30	0.319
WBC, × 10^9^/L	8.57 ± 5.37	9.05 ± 6.10	7.78 ± 3.87	0.284
NLR	8.30 ± 11.64	10.13 ± 14.14	5.32 ± 4.37	0.040*
PLT, × 10^9^/L	226 ± 106	235 ± 113	211 ± 94	0.335

Data are presented as the means ± standard deviation or interquartile range. *p < 0.05, **p < 0.01, and ***p < 0.001. *p* values were determined by Student’s *t* test for continuous variables and the chi-square test for categorical variables

*Abbreviations*: *LDH* Lactate dehydrogenase, *HCY* Homocysteine, *FIB* Fibrinogen, *CEA* Carcinoembryonic antigen, *AFP* Alpha-fetoprotein, *TP* Total protein, *ALB* Albumin, *Ca* Total calcium, *WBC* Leukocyte, *NLR* Neutrophil-to-lymphocyte ratio, *PLT* Platelets

Reference ranges: α-HBDH: 95–220U/L, LDH: 109–245U/L, HCY: 5–20umol/L, FIB: 2–4 g/L, CEA: 0–5.5 ng/ml, AFP: 0–6.05 IU/ml, TP: 60–82 g/L, ALB: 35–50 g/L, Ca: 2–3 mmol/L, WBC: 4–10 × 10^9^/L, PLT: 100–300 × 10^9^/L

Based on α-HBDH level, of the 71 patients, 27 were assigned to the normal-α-HBDH group (≤ 220 U/L), while 44 were assigned to the high-α-HBDH group (> 220 U/L). Table 1 Statistical calculation results showed that there were statistically significant differences in gender (*P* = 0.021) and lymph node metastasis (*P* = 0.042) time between the high-α-HBDH group and the normal-α-HBDH group in general clinical data. LDH (*P* < 0.001) and NLR (*P* = 0.04) were statistically different between the two groups.

### α-HBDH was an independent factor for overall survival in univariate and multivariate cox regression

As shown in Table 2, the univariate analysis found that age, liver metastasis, α-HBDH, ALB, WBC, and NLR were associated with overall survival with a statistical difference (*P* < 0.05). In order to include as many clinical indicators as possible, we included all parameters of *P* < 0.1 in the multivariate cox analysis, the result showed that age, liver metastasis, α-HBDH, and NLR levels were still major factors affecting survival.

**Table 2:** Univariate and multivariate analysis of clinical characteristics and laboratory data of in LC patients

	Univariate analysis			Multivariate analysis		
	*p*	HR	95% Cl	*p*	HR	95%Cl
Age	**0.004**	2.168	1.266–3.713	0.018*	1.966	1.123–3.440
Gender	0.173	1.485	0.839–2.629			
Smoking	0.258	1.337	0.807–2.213			
Metastasis						
Liver	**0.002**	3.278	1.501–7.161	0.011*	2.911	1.277–6.636
Lymphnodes	0.471	1.193	0.738–1.926			
Kidney	0.356	0.519	0.125–2.146			
Brain	0.172	0.645	0.342–1.216			
Bone	0.505	1.199	0.703–2.042			
Staging	0.754	1.044	0.799–1.363			
Classification	0.680	1.080	0.749–1.556			
α-HBDH (U/L)	**0.023**	1.774	1.075–2.926	0.015*	1.892	1.129–3.170
LDH (U/L)	**0.068**	1.559	0.964–2.521	0.895		
HCY (umol/L)	0.299	1.288	0.798–2.080			
FIB g/L	**0.075**	1.551	0.954–2.522	0.151		
CEA ng/ml	**0.062**	0.250	0.058–1.072	0.933		
AFP IU/ml	0.170	0.655	0.356–1.204			
TP g/L	0.227	0.687	0.372–1.268			
ALB g/L	**0.021**	0.556	0.336–0.922	0.566		
A/G	0.368	0.780	0.453–1.342			
Ca mmol/L	0.671	0.832	0.355–1.947			
WBC × 10^9^/L	**0.013**	1.896	1.144–3.145	0.153		
NLR	**0.009**	1.965	1.174–3.290	0.031*	1.787	1.054–3.029
PLT × 10^9^/L	0.629	0.894	0.567–1.409			

*HR* Hazards ratio

^*^p < 0.05, **p < 0.01, and ***p < 0.001

### ROC of α-HBDH and LDH for LC

**Fig. 1:**
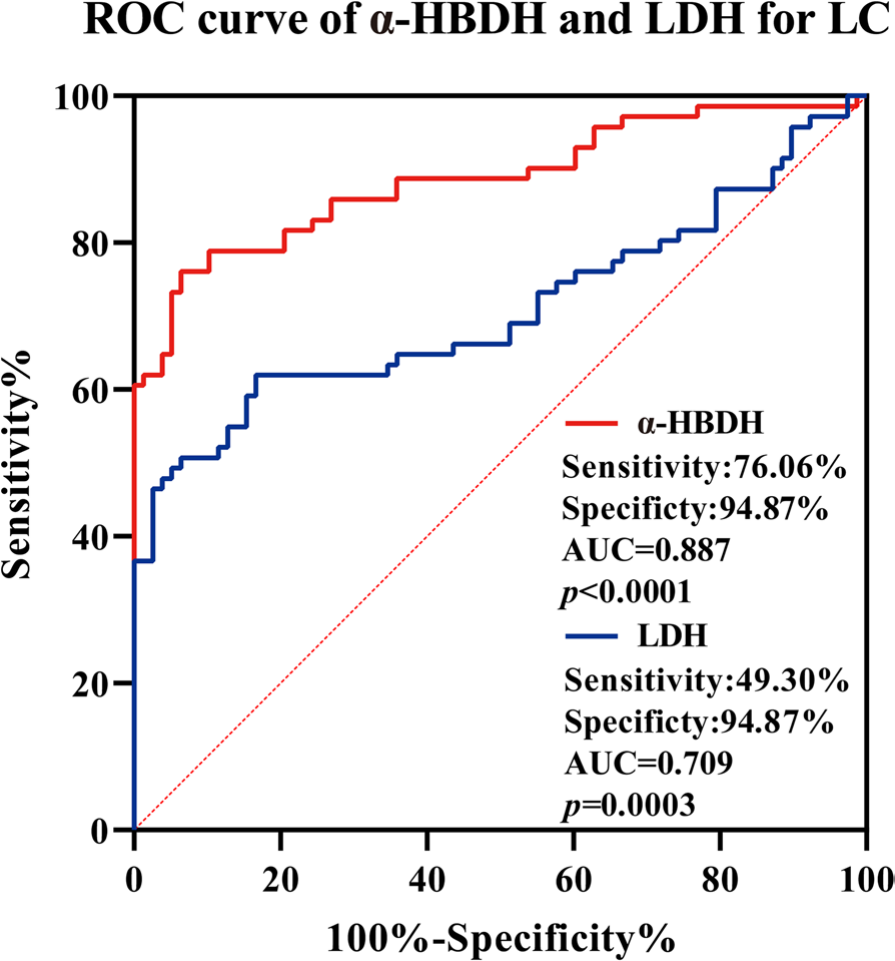
ROC curve of α-HBDH and LDH for LC

In addition, ROC analysis of LC showed a higher AUC value in α-HBDH (AUC = 0.887, 95%Cl 0.827–0.947, *P* < 0.0001) than LDH (AUC = 0.709, 95%Cl 0.615–0.803, *P* = 0.0003). The optimal clinical cutoff value of α-HBDH for distinguishing LC patients was 197 U/L, which provided a 76.06% sensitivity and a 94.87% specificity, which was higher than the sensitivity (49.30%) of LDH. The results showed a higher overall diagnostic efficacy of α-HBDH than LDH (Fig. 1).

### OS of α-HBDH and LDH for LC

**Fig. 2:**
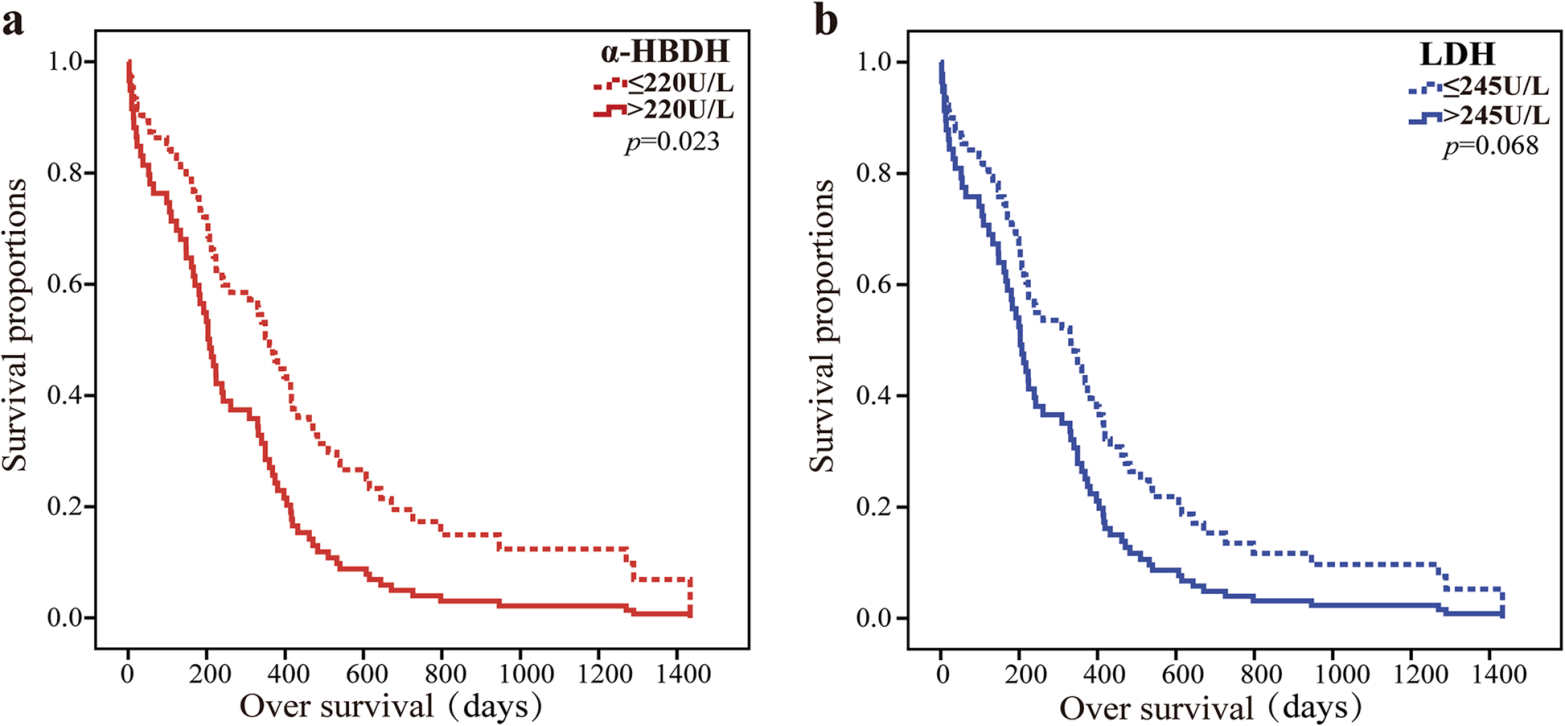
OS of α-HBDH (**a**) and LDH (**b**) for LC

In this study, compared with the normal group of α-HBDH (12.7 months, 95%Cl 0.8–46.4), the group of high-α-HBDH had a significantly lower median OS (6.4 months, 95%Cl 0.2–26.0), and the difference was statistically significant (*P* = 0.023). The median OS of the LDH > 245 U/L group was 5.8 (95%Cl 0.1–34.4) months, and the LDH ≤ 245 U/L group was 12.0 (95%Cl 0.3–44.7) months; however, the difference was not statistically significant (*P* = 0.068) (Fig. 2a, b).

## Discussion

Lung cancer (LC) is one of the most common malignant tumors with high morbidity and mortality and seriously endangers human life and health [20]. Early assessment of the severity of LC is a key factor in determining treatment strategies. At present, the diagnosis of LC is mainly based on low-dose spiral CT and serological tumor markers [21]. Some serological tumor markers have poor sensitivity and specificity, so we need to find more biomarkers to improve the diagnostic efficacy and make a more accurate judgment of the severity and prognosis of the disease. Usually, serum α-HBDH and LDH are common biochemical index in the laboratory, and α-HBDH was generally detected by the α-ketobutyrate method and LDH was lactate method, which was simple and quick detection method by using a fully automated biochemical analyzer. In this study, we found that the level of α-HBDH was elevated easier compared with the other conventional biochemical indexes in LC patients. This is consistent with some studies in intracranial and testicular tumors [12, 13, 19]. This indicates that the serum level of this index is also abnormally elevated in patients with lung cancer.

The male-to-female ratio in this cohort was nearly 2.73, which is 1.5 times the world average for lung cancer incidence [22]. We also found a significantly higher proportion of males in the normal-α-HBDH group and the high-α-HBDH group, and the male with a female ratio was significantly different (*χ*^*2*^ = 4.232, *P* = 0.021). Since this study is a retrospective study and there are standard screening cases, there may be biases such as selection bias and confounding bias leading to errors, and a large sample is still needed to avoid these biases. However, α-HBDH levels were not affected by the higher proportion of men in the normal-α-HBDH group. The result showed that smoking also was not affected the level of α-HBDH, So the difference in gender between the two groups did not significantly affect the study results.

In this study, among all 33 cases with lymph node metastasis, 24 cases had a high serum value of this index, accounting for 55% of all high-value cases. This indicates that the elevation of α-HBDH is probably associated with lymph node metastasis. The reason may be that in the tumor microenvironment, the glycolysis of malignant tumor tissues is higher than that of normal tissues, and tumor cells preferentially use lactic acid as an energy source, which reduces the pH value in vivo and promotes the invasion and metastasis of tumor cells [23]. Scholars studied that elevated serum LDH has the potential to predict early metastasis, according to an analysis of serum biomarkers in 267 patients with gastrointestinal cancer [24]. As we all know, lymph node metastasis is associated with poor prognosis, which indirectly suggests that elevated lymph node metastasis may be associated with poor prognosis.

In the serum of small-cell carcinoma patients, it was determined that the enzymes LDH and α-HBDH were shown with the highest share of abnormal values by Warnecke et al. study [14]. Compared with the laboratory data of the two groups, there was a correlation between this index and LDH, and the value of LDH in the high-α-HBDH group was significantly higher than that in the normal-α-HBDH group. This indicates that these two indexes have similar effects, and the poor prognosis of other tumors may be negatively correlated. Consistent with the results of this study, Khanolkar et al. found that serum LDH and α-HBDH levels were elevated in both seminomatous and non-seminomatous germ cell tumors in 1990, and α-HBDH was being more specific in monitoring therapy as compared to serum LDH [19].

NLR is the ratio of neutrophil to lymphocyte counts in serum, which can be used as a prognostic indicator in inflammation and some tumors [25]. It is also a putative measure of the balance between neutrophil-associated tumor-pro-inflammatory response and lymphocyte-dependent antitumor immune function and has been proposed as a prognostic factor in a variety of cancers [25, 26]. In this study, the serum NLR value of patients in the high-α-HBDH group was significantly higher than that in the normal-α-HBDH group, indicating that the prognosis of patients in the high-value group was poor. α-HBDH is not an independent specific enzyme, but rather the LDH isoenzyme total term of LDH-1 and LDH-2 containing the H subunits. Metabolic changes in rapidly dividing cancer cells are closely associated with increased uptake of glucose and abnormal activity of LDH, which regulates the processing of glucose to lactic acid [27, 28]. The authors suggest a possible mechanism which is the α-HBDH, expressed by malignant cells, significantly increases lactate formation, and lactate induces the proliferation of oxygenated malignant cells and angiogenesis and inhibits the innate and adaptive immune responses [29]. This process results that α-HBDH and LDH activities were elevated in malignant cells, and the value of NLR was downgraded in serum.

The prognosis and 5-year survival of cancer patients may be influenced by some factors, such as tumor classification, early postoperative chemotherapy [30, 31], and metastasis. In the survival analysis of this study, it was confirmed that the elevated α-HBDH levels, NLR, WBC, liver metastasis, and age were the risk factors affecting OS, and the association was positive between α-HBDH and the increased risk of OS (HR 1.892, 95%Cl 1.129–3.170, *P* = 0.015). Studies have shown that elevated α-HBDH in some acute heart and lung diseases suggests poor survival [8, 32]. As in other diseases, an increase in this index predicted poor overall survival in LC patients. So we have explored the correlation between α-HBDH elevation and the prognosis of LC and found that baseline serum α-HBDH elevation correlates with shorter survival, and α-HBDH can be identified as an independent risk factor for LC.

LDH has been incorporated into the Ranson score system to assess tumor severity and predict cancer prognosis. Currently, elevated serum LDH in solid tumors is associated with clinical outcomes in a variety of cancers, and tumor prognosis is a risk factor for outcomes [17, 33]. Sun et al. found that serum LDH and α-HBDH levels were significantly increased in patients with malignant tumors, and these levels can be used as reference indicators for the diagnosis and treatment of digestive system malignant tumors [34]. The result showed that the high-α-HBDH group was more significant in predicting OS than the high LDH group (0.023 vs 0.068). In addition, ROC analysis showed that α-HBDH had a predictive value (AUC = 0.887) better than LDH (AUC = 0.709) for LC, mainly manifested in α-HBDH has a high sensitivity value (76.06% vs 49.30%) than LDH. This is perhaps the reason why we found that α-HBDH is more valuable than LDH in LC.

In summary, as serum α-HBDH levels were found to be commonly increased in LC patients and correlated with poor clinical outcomes in this study. The determination of α-HBDH may become a supportive tool in prognosis cancers. This study excluded other factors and proved the important value of serum α-HBDH in the prognosis of lung cancer, but there are still some deficiencies in this study. First of all, the amount of data in this study is small. This may be because α-HBDH is not tested as a routine testing indicator, especially in tumor patients with the non-cardiac disease. Secondly, the data of this study came from a single center, and there may be biases such as selective and confounding bias leading to errors, and a large sample is still needed to avoid these biases. What is more, the AUC difference between models. The AUC has been criticized for its insensitivity in model comparisons where the baseline model is better, and NRI (net reclassification improvement) and IDI (integrated discrimination improvement) have been proposed as alternatives to the increase in the AUC for evaluating improvement in the performance of risk assessment algorithms. However, NRI and IDI are also not without problems [35]. In addition, this study was a retrospective prognostic study and the prognostic validation set also took longer, and it was not completed in the short term. In the future, a multi-center study of medical record tracking will be more helpful to prove whether α-HBDH can be used as a basis for the prognosis of lung cancer. So, a single serological laboratory index cannot fully be a key evaluation index of the tumor prognosis, but it can provide a powerful auxiliary tool for disease severity and prognosis for LC patients.

## Conclusion

In conclusion, this study suggested that α-HBDH is an independent risk factor for poor prognosis in LC patients. And also showed that serum α-HBDH was more prognostic sensitive than LDH.

## Supplementary Information

**Additional file 1:** **Figure 1.** ROC curve of α-HBDH and LDH for LC. **Figure 2.** OS of α-HBDH (a) and LDH (b) for LC.

## Data Availability

The datasets used and/or analyzed during the current study are available from the corresponding author on reasonable request.
