# Supplementary material for: Prognostic value of serum α-HBDH levels in patients with lung cancer
Source: World J Surg Oncol. 2023 Mar 6;21:78. doi: 10.1186/s12957-023-02965-3 (PMC9987145; doi:10.1186/s12957-023-02965-3)
Supplement: Supplementary file 1 — Additional file 1: Figure 1. ROC curve of α-HBDH and LDH for LC. Figure 2. OS of α-HBDH (a) and LDH (b) for LC. [file 12957_2023_2965_MOESM1_ESM.docx]

**Figure 1** **ROC curve of α-HBDH and LDH for LC**

**Figure 2** **OS of α-HBDH (a) and LDH (b) for LC**
